# Typhoid Perforation1From *Louisville Monthly Journal of Medicine and Surgery*, March, 1904.

**Published:** 1904-06

**Authors:** Louis Frank

**Affiliations:** Louisville, Ky.


### SELECTIONS.

### Typhoid Perforation.[1]

By LOUIS FRANK, M. D., Louisville, Ky.

IT IS only necessary to make a cursory view of the medical and surgical literature of the day to be impressed with the importance of the proper treatment of intestinal perforations occurring as a complication of typhoid fever. The advances in therapeutics have robbed typhoid of many of its dangers, until today under these improved methods of treatment, the mortality has been reduced to a little less than 10 per cent. Can this be lowered we ask ourselves? We think yes, in view of the fact that about 3 per cent. of all cases die of perforation. This means one-third of all the deaths in this disease is from the peritonitis consequent upon the escape of intestinal contents. We believe that one-half of such deaths could be prevented by proper surgical intervention.

The greatest stimulus was given to the surgical complications of typhoid by the classical monograph of Prof. Keen who at the time his work was issued, (and one, by the way, which every practitioner should read) was able to collect only 83 cases upon which an operation had been done to save the life of the patient. Of these 83, 19.3 per cent. recovered.[2] One year later 47 more

1. From *Louisville Monthly Journal of Medicine and Surgery*, March, 1904.
2. The Surgical Complications and Sequelæ of Typhoid Fever, by W. W. Keen.

cases had been operated, and the death rate had diminished considerably as 28 per cent. got well. In September, 1903, the writer was able to collect 339 cases with 90 recoveries.

One may say that this is little improvement. True, but as answer to this we can say that formerly these 90 cases would all have died and that many of these cases were operated upon even late after the perforation had taken place.

This danger is one that is always present, that we can never foresee, that we can not avoid, one which is likely to occur in the very mildest case as in the severest and which is at the same time, if not recognised at once and properly handled, the most fatal of any complication or accident in the course of the disease. Without surgical aid recovery from such perforation is so rare that we may say it practically does not occur. Admitting this we must conclude that we should endeavor (1) to recognise the very earliest manifestations of this accident, i. e., to make an early and correct diagnosis, and having done so, (2) to give our patient the advantage of proper surgical assistance. Early recognition and proper surgical aid go hand in hand, the one without the other is useless. Neither, however, should be difficult, and, in this day of surgery when good surgeons are found within a few hours distant from most all and every locality, there is hardly an excuse for not having the latter and we believe the former, that is, the early recognition, is within the possibilities of every practitioner if the proper interpretation is put upon the symptoms.

The symptom complex is due to the peritoneal insult and manifests itself as soon as this structure is involved. The important ones are pain and tenderness and rigidity.

Pain in the belly, especially in the right iliac region coming on suddenly or sharply during the course of typhoid, either in the first or consequent weeks, and not relieved after an enema or spontaneously in a very short time is a most grave symptom and one which usually means something. Should this be accompanied by tenderness in the same region and should there be rigidity, gradually extending over the abdomen, the diagnosis, even in the absence of all other symptoms, should be one of probable perforation. Many errors have arisen on account of the textbook descriptions of the symptomatology, in which is pictured so graphically and strikingly the symptom of shock as a necessary accompaniment of perforation. As some one has said, "these descriptions and symptoms are the best ever written of impending death and are not at all those of the perforation."

Shock is not a symptom in many cases. Shock comes on often very late, too late to do good by any form of treatment.

Shock should not be awaited either before making a diagnosis nor as an indication for operation. On the other hand it is no bar nor is it always a contraindication to operation.

Of the other symptoms, pulse, temperature, vomiting, distention, tympany over the area of normal liver dulness and altered facial expression are all too inconstant and too variable to be depended upon. The three always present, the cardinal symptoms, the all sufficient indices, are pain, tenderness and rigidity, or, in the order of importance probably rigidity, pain, tenderness The manifestation of any one of these during the course of the disease is cause to redouble or treble one's vigilance, making frequenter visits or remaining with the patient until satisfied as to the cause for its production, and at the same time we would get in touch with the surgeon, if necessary have him see the case in consultation, because we believe he is able to more readily recognise peritoneal damage than those not accustomed to deal with these structures, nor so familiar with their pathology.

Robert Morris recently expressed himself as believing that in every case of typhoid the physician should have a surgical consultant ready for just such emergencies. With such an arrangement the mortality should be greatly reduced and at least one-half of the cases now dying from this cause could and would be saved. It is largely on account of early recognition and prompt surgery, even in doubtful cases, that so many more are saved in hospital than in private practice, and this will, to a certain extent, always be true. Much can, however, be done in general practice if there is a thorough appreciation of the constant danger and the early symptoms and a proper understanding and relationship established between physician and surgeon.

A few words as to the surgical indications and technic: the best results are obtained in cases operated within the first twelve hours after rupture. Of cases operated upon within this period between 50 and 60 per cent. have recovered. The next most favorable period is within 24 hours; after 36 hours have elapsed most all cases will die, operation or no operation.

The incision should be made in the right linea semilunaris, as the vast majority of perforations occur within the last 12 inches of the ileum.

After the abdomen is opened search at once for the cecum and reach the distal 12 or 20 inches of the gut. When the perforation is found, waste no time trimming the ulcer or in fancy work, but close the opening, either by a purse string suture or by a continuous right angle suture, placed horizontally to the long axis of the gut. If only one opening is found it may be stitched to the parietal wound. Wash out the cavity rapidly and thoroughly with physiological saline solution and close the abdomen, after providing ample and abundant drainage.

If general anesthesia is feared use cocaine locally, but waste no time in fancy frills or surgical refinements. These latter are dangerous, but even such are not as dangerous as delay in diagnosis.

In conclusion, we would add the following summary of a paper recently read by the writer before the Mississippi Valley Medical Association:

1. Perforations are to be expected in about 2.5 per cent. of all cases of typhoid fever.

2. Prompt surgical intervention is the best and only logical treatment.

3. Early diagnosis is most desirable and will be the means of greatly reducing the mortality, as 55 to 60 per cent. would recover.

4. Diagnosis, sufficiently early to achieve these results, can only be made by careful watching, treating all cases as serious and a proper interpretation of the symptoms. At the first indication have the surgeon in consultation and be prepared to operate.

5. More cases die from delay than errors in surgical technic, therefore in doubtful cases, though a mistake may be made and no perforation found, operate.

6. No case, unless dying, is so desperate as to be beyond some hope of saving, so in operating be rapid, lose no time in fancy work or in surgical refinement and be sure to drain.

7. "Get into the belly quickly and get out more quickly."

